# Modulation of nuclear factor-kappa B activation by the endoplasmic reticulum stress sensor PERK to mediate estrogen-induced apoptosis in breast cancer cells

**DOI:** 10.1038/s41420-017-0012-7

**Published:** 2018-02-12

**Authors:** Ping Fan, Amit K. Tyagi, Fadeke A. Agboke, Rohit Mathur, Niranjana Pokharel, V. Craig Jordan

**Affiliations:** 1 Department of Breast Medical Oncology, The University of Texas MD Anderson Cancer Center, Houston, TX USA; 2 Department of Oncology, Lombardi Comprehensive Cancer Center, Georgetown University, Washington, DC USA; 3 Department of Lymphoma/Myeloma, The University of Texas MD Anderson Cancer Center, Houston, TX USA

## Abstract

Stress responses are critical for estrogen (E_2_)-induced apoptosis in E_2_-deprived breast cancer cells. Nuclear factor-kappa B (NF-κB) is an important therapeutic target to prevent stress responses in chronic inflammatory diseases including cancer. However, whether E_2_ activates NF-κB to participate in stress-associated apoptosis in E_2_-deprived breast cancer cells is unknown. Here, we demonstrated that E_2_ differentially modulates NF-κB activity according to treatment time. E_2_ initially has significant potential to suppress NF-κB activation; it completely blocks tumor necrosis factor alpha (TNFα)-induced activation of NF-κB. We found that E_2_ preferentially and constantly enhances the expression of the adipogenic transcription factor CCAAT/enhancer binding protein beta (C/EBPβ), which is responsible for the suppression of NF-κB activation by E_2_ in MCF-7:5C cells. Interestingly, NF-κB p65 DNA-binding activity is increased when E_2_ is administered for 48 h, leading to the induction of TNFα and associated apoptosis. Blocking the nuclear translocation of NF-κB can completely prevent the induction of TNFα and apoptosis induced by E_2_. Further examination revealed that protein kinase RNA-like endoplasmic reticulum kinase (PERK), a stress sensor of unfolded protein response (UPR), plays an essential role in the late activation of NF-κB by E_2_. This modulation between PERK and NF-κB is mainly mediated by a stress responsive transcription factor, transducer and activator of transcription 3 (STAT3), independently of the classic canonical IκBα signaling pathway. Thus, inhibition of PERK kinase activity completely blocks the DNA binding of both STAT3 and NF-κB, thereby preventing induction of NF-κB-dependent genes and E_2_-induced apoptosis. All of these findings suggest that PERK is a key regulator to convey stress signals from the endoplasmic reticulum to the nucleus and illustrate a crucial role for the novel PERK/STAT3/NF-κB/TNFα axis in E_2_-induced apoptosis in E_2_-deprived breast cancer cells.

## Introduction

Targeting the estrogen receptor (ER) with a selective estrogen receptor modulator (SERM) or inhibiting synthesis of estrogen (E_2_) with an aromatase inhibitor are successful therapeutic strategies to treat or prevent ER-positive breast cancer^1^. However, acquired resistance to anti-hormone therapies will inevitably occur for the majority of treated patients. Paradoxically, the discoveries that physiological levels of E_2_ can induce regression of SERM-resistant breast tumors in athymic mice^2, 3^ and induce apoptosis in E_2_-deprived breast cancer cells^4, 5^ have resulted in a novel therapy in breast cancer patients following exhaustive anti-hormone therapy^6^. This was the scientific rationale behind the use of E_2_ to treat aromatase inhibitor-resistant breast cancer in clinical trials with 30% benefit for patients^7^. Furthermore, hormone replacement therapy (HRT) with only E_2_ in postmenopausal women in their 60s has a reduced incidence of breast cancer and mortality^8^ because of E_2_-induced apoptosis^6^, whereas classic HRT with E_2_ plus medroxyprogesterone acetate (MPA) increases the risk of breast cancer^8^. This is because MPA acts like a glucocorticoid to block E_2_-induced apoptosis^9^. All of these clinically relevant findings encouraged us to investigate the mechanism underlying E_2_-induced apoptosis and identify the key checkpoints involved, so that the therapeutic effects of E_2_ in anti-hormone therapy-resistant breast cancer can be enhanced.

Unlike rapid chemotherapy-induced apoptosis, E_2_ induces apoptosis in a delayed manner, with initial cellular proliferation in response to E_2_ exposure in E_2_-deprived breast cancer cells^11, 10^. Our recent investigations revealed that accumulation of stress responses, including endoplasmic reticulum, oxidative, and inflammatory stresses, results in E_2_-induced apoptosis^12, 11^. The endoplasmic reticulum is a crucial regulatory site for stress responses^13^. Three stress sensors of unfolded protein response (UPR), protein kinase RNA-like endoplasmic reticulum kinase (PERK), inositol-requiring protein 1 alpha (IRE1α), and activating transcription factor 6 (ATF-6) are initially activated by E_2_ as an adaptation response to maintain homeostasis in the endoplasmic reticulum^15, 11, 14^. PERK phosphorylates eukaryotic initiation factor 2 alpha (eIF2α) to attenuate protein translation^17, 16^ which is identified as an important mediator of E_2_-induced apoptosis^11^, whereas ATF-6 and IRE1α are involved in endoplasmic reticulum-associated protein degradation (ERAD) of phosphoinositide 3-kinase (PI3K)/Akt/mTOR-related pathways^13^. Additionally, a variety of stress- and inflammation-responsive genes, such as tumor necrosis factor alpha (TNFα), lymphotoxin alpha (LTA), lymphotoxin beta (LTB), and interleukin-6 (IL-6), are activated to create a special inflammatory microenvironment after E_2_ exposure^12, 11^. Among these inflammatory factors, the function of TNFα has been confirmed to be an important factor to induce apoptosis with higher levels of cleaved PARP and caspase 9 in MCF-7:5C^11^. Induction of TNFα by E_2_ reaches a peak at 3 days in MCF-7:5C cells, whereas the highest levels of TNFα occur after 9–12 days of E_2_ treatment in MCF-7:2A cells^18, 12^. In line with the time point of TNFα induction, E_2_-induces apoptosis in MCF-7:5C cells within 1 week, while apoptosis is delayed to 2 weeks after exposure to E_2_ in MCF-7:2A cells^18, 11^. Nevertheless, how E_2_ induces TNFα and why a delay occurs still need to be explained.

It is well known that TNFα is a nuclear factor-kappa (NF-κB)-dependent gene; on the other hand, TNFα is also a strong inducer for NF-κB^19^. However, it remains unknown whether E_2_ induces TNFα via activation of NF-κB in E_2_-deprived breast cancer cells. There is cross-talk existing between ER and NF-κB, but the latter is repressed by ER in breast cancer^20^. Nonetheless, NF-κB can be activated in the setting of endoplasmic reticulum stress even though the mechanisms are poorly understood^19^. Furthermore, compelling evidence indicates that NF-κB and lipid metabolism-associated transcription factor C/EBPβ can interact to modulate endoplasmic reticulum stress and inflammatory responses^22, 21^. Thus, NF-κB has proven to be a viable therapeutic target for diseases related to endoplasmic reticulum stress, such as neurodegenerative diseases and diabetes^23, 24^. In most quiescent cells, NF-κB binds to the inhibitory IκB proteins in the cytosol and forms an inactive complex. A common canonical pathway that activates NF-κB is modulated by the degradation of IκB proteins, which leads to the release of NF-κB from the complex and translocation to the nucleus where NF-κB-dependent genes, such as TNFα and LTB, are activated^19^. In addition, post-translational modifications of NF-κB protein subunits by kinases play important roles in the activation of the NF-κB response^25^. Under endoplasmic reticulum stress, a downstream signal of PERK, eIF2α has been reported to be sufficient to activate NF-κB DNA binding through decreasing levels of IκBα, but does not affect the stability of the protein^25^. We observed that E_2_ activates PERK/eIF2α^11^, but there are no reports concerning the interaction between PERK and the activation of NF-κB in modulation of E_2_-induced apoptosis in E_2_-deprived breast cancer cells.

We sought here to further investigate how PERK kinase activates NF-κB/TNFα axis to affect E_2_-induced apoptosis. Our data demonstrate that E_2_ initially suppresses activation of NF-κB and effectively prevents TNFα from activating NF-κB. We note a preferential elevation of C/EBPβ expression by E_2_ that is responsible for the suppression of NF-κB. However, NF-κB is subsequently activated by PERK via activation of transcription factor STAT3. This is independent of eIF2α phosphorylation and the canonical IκBα signaling pathway. Blockade of PERK phosphorylation and treatment with a NF-κB inhibitor produce equivalent effects in preventing E_2_ from inducing TNFα expression and apoptosis. In addition to regulating the function of STAT3 and NF-κB, PERK modulates multiple stress responsive transcription factors, including nuclear factor erythroid-derived 2-like 2 (Nrf2), hypoxia-inducible factor 1-alpha (HIF-1α), and ERα-target genes, after E_2_ treatment. This suggests that PERK is critical to convey stress signals from the endoplasmic reticulum to the nucleus with multiple stress-responsive transcription factors involved. Together, this study provides an important rationale for the exploration of targeting the stress responses in breast cancer patients undergoing exhaustive anti-hormone therapy.

## Results

### NF-κB is constitutively activated in MCF-7:5C cells

**Fig. 1:**
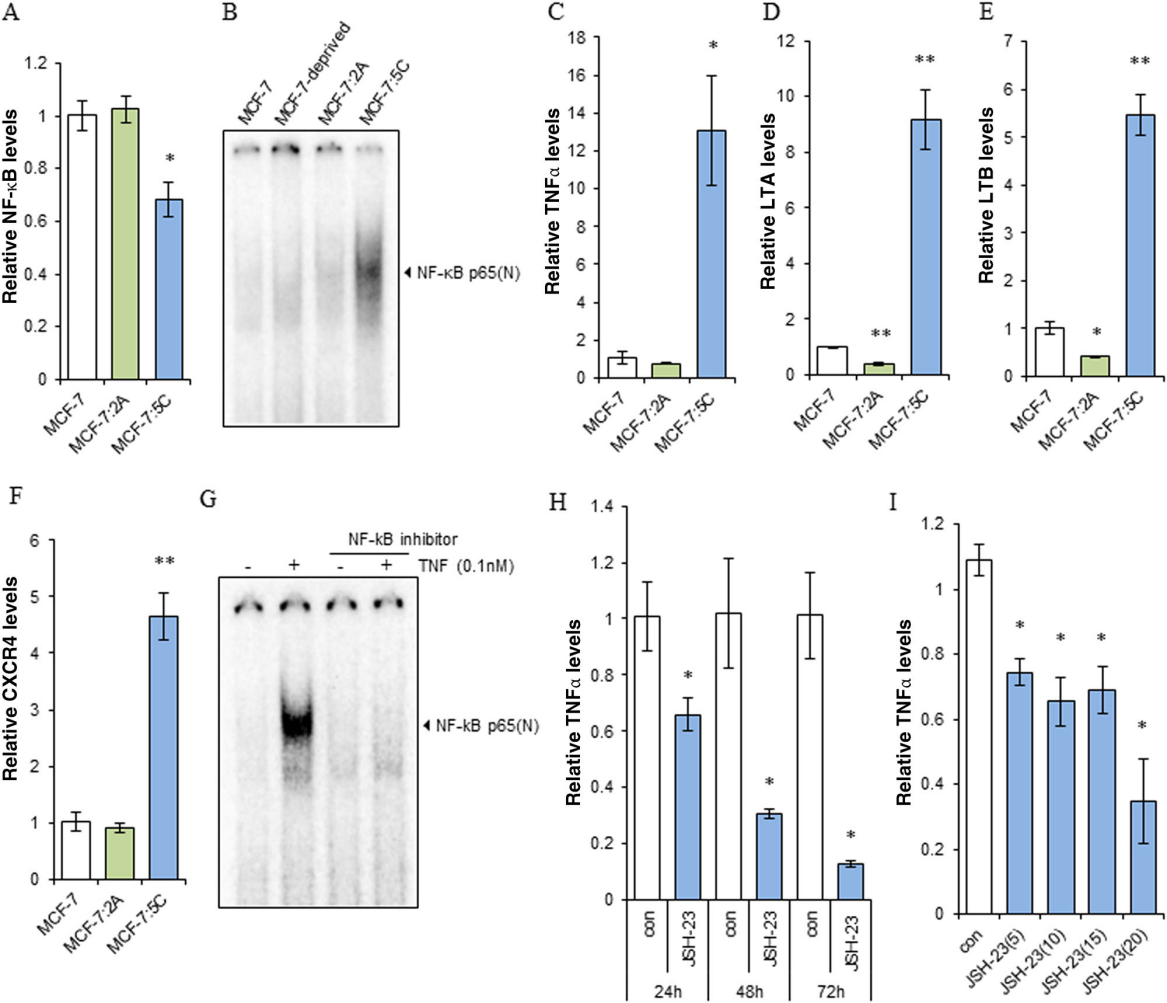
NF-κB is constitutively activated in MCF-7:5C cells. **a** MCF-7 cells were transferred to E_2_-free medium for 3 days. Cells were harvested in Trizol similarly to that with the E_2_-deprived cell lines MCF-7:5C and MCF-7:2A. NF-κB expression was quantitated by RT-PCR. *P < 0.05 compared with MCF-7 cells. **b** NF-κB DNA-binding activity. MCF-7 cells were cultured in parallel in E_2_-replete or E_2_-depleted medium for 3 days. Next, cells were harvested similarly to that with MCF-7:5C and MCF-7:2A for EMSA. **c**−**f** Basal mRNA expression levels of TNFα (**c**), LTA (**d**), LTB (**e**), and CXCR4 (**f**) in three cell lines were quantitated by RT-PCR as in **a**. *P < 0.05 and **P < 0.001 compared with MCF-7 cells. **g** The NF-κB inhibitor blocked NF-κB/DNA-binding. MCF-7:5C cells were treated with TNFα (0.1 nm), the NF-κB inhibitor JSH-23 (20 µm), or a combination of them for 30 min for EMSA. **h** MCF-7:5C cells were treated with JSH-23 (10 µm) for 24, 48, and 72 h. TNFα expression levels were quantitated by RT-PCR. *P < 0.05 compared with control. **i** MCF-7:5C cells were treated with different concentrations (5, 10, 15, 20 µm) of JSH-23 for 48 h. TNFα expression levels were quantitated by RT-PCR. *P < 0.05 compared with control

Many genes are activated during the stress response caused by E_2_ deprivation^13^. To determine whether E_2_ deprivation affects the basal levels or activation of NF-κB, we compared the E_2_-deprived breast cancer cell lines MCF-7:5C and MCF-7:2A with parental MCF-7 cells. MCF-7:5C cells had lower expression levels of NF-κB than did the other two cell lines. MCF-7:2A and MCF-7 cells had similar NF-κB expression levels (Fig. 1a). However, MCF-7:5C had higher levels of constitutively activated NF-κB than did the other cell lines (Fig. 1b). We detected this difference using electrophoretic mobility shift assays (EMSA), an appropriate way to assess the nuclear activation of NF-κB via direct NF-κB p65 DNA binding^26^. The higher levels of active NF-κB in MCF-7:5C cells resulted in higher basal expression levels of NF-κB-dependent genes, e.g. TNF family members (TNFα, LTA, and LTB) and the chemokine receptor CXCR4 (Fig. 1c, d, e, f). Treatment with the selective NF-κB inhibitor JSH-23 effectively prevented nuclear translocation of NF-κB and completely blocked NF-κB p65 DNA binding induced by TNFα, which is a strong inducer of NF-κB activation (Fig. 1g). JSH-23 also effectively reduced the expression of TNFα and LTB in a time- and dose-dependent manner (Fig. 1h, i and Supplementary Fig. S1A). Importantly, it also markedly inhibited the proliferation of the three breast cancer cell lines after different concentrations treatment (Supplementary Fig. S1C−1E). These results indicated that long-term E_2_ deprivation may alter the activation of NF-κB, which acts as a growth signal in breast cancer cell lines.

### E_2_ rapidly suppresses nuclear activation of NF-κB in E_2_-deprived breast cancer cells

**Fig. 2:**
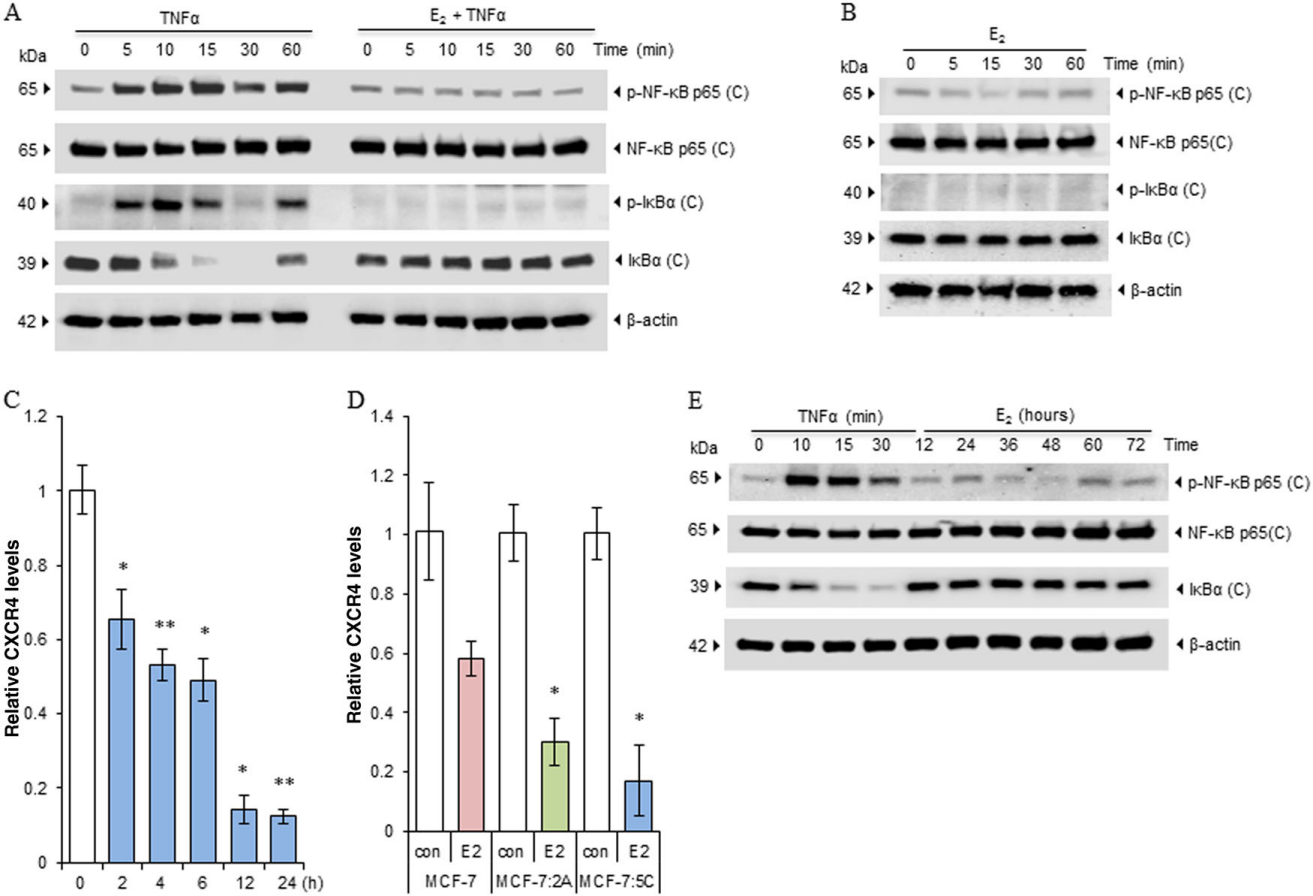
E_2_ suppresses activation of NF-κB in MCF-7:5C cells. **a**,** b** MCF-7:5C cells were treated with TNFα (0.1 nm), E_2_ (1 nm), or a combination of them for the indicated periods. Cells were then harvested to isolate cytosol fractions for immunoblotting with different antibodies against phosphorylated or total NF-κB and IκBα. **c** MCF-7:5C cells were treated with E_2_ (1 nm) for the indicated periods. CXCR4 expression levels were quantitated by RT-PCR. *P < 0.05 and **P < 0.001 compared with control. **d** MCF-7 cells were transferred to E_2_-free medium for 3 days. Next, MCF-7, MCF-7:5C, and MCF-7:2A cells were treated with E_2_ for 24 h. Cells were harvested in Trizol for mRNA extraction. CXCR4 expression levels were quantitated by RT-PCR. *P < 0.05 compared with control. **e** MCF-7:5C cells were treated with TNF (0.1 nm) or E_2_ (1 nm) for the indicated periods. Cells were then harvested to isolate cytosol fractions for immunoblotting with different antibodies against phosphorylated or total NF-κB and IκBα

To further determine whether E_2_ regulates the activation of NF-κB, we first treated MCF-7:5C cells with E_2_ for different periods, using cells treated with TNFα as positive controls. As expected, TNFα quickly increased NF-κB phosphorylation after 5 min of treatment. Simultaneously, the classic canonical signaling was activated, demonstrating increased levels of phosphorylated IκBα and higher degradation rate of IκBα, as compared with control. E_2_ had a significant potential for completely suppressing TNFα-induced activation of NF-κB and IκBα (Fig. 2a). Unlike TNFα treatment, E_2_ alone did not significantly change these signaling pathways within 1 h (Fig. 2b). In agreement with this, expression of NF-κB target gene *CXCR4* was suppressed by E_2_ in MCF-7:5C cells (Fig. 2c), as well as in both MCF-7 and MCF-7:2A cells (Fig. 2d). Treatment with E_2_ also reduced the expression levels of NF-κB in MCF-7:5C cells, but not in the other two cell lines (Supplementary Fig. S1B). We detected a weak elevation of phosphorylated NF-κB p65 in the cytosol of MCF-7:5C cells when treated with E_2_ for more than 48 h (Fig. 2e), while we did not observe elevated phosphorylation or detectable degradation of IκBα in parallel (Fig. 2e), indicating that other mechanisms are involved in the regulation of NF-κB activation by E_2_.

### Preferential elevation of C/EBPβ expression induced by E_2_ is responsible for suppression of NF-κB activity

**Fig. 3:**
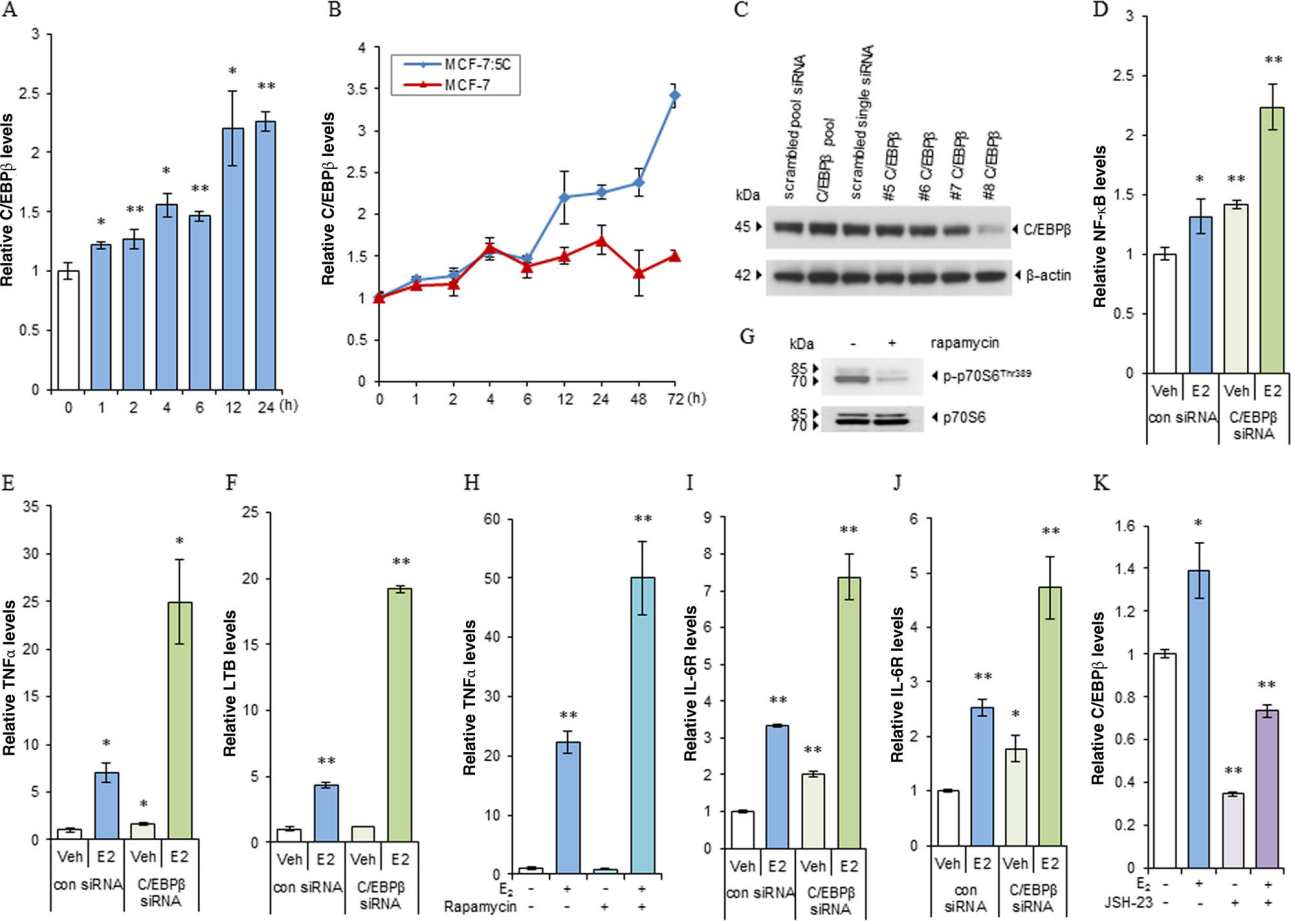
E_2_ preferentially elevates C/EBPβ expression to suppress the function of NF-κB. **a** MCF-7:5C cells were treated with E_2_ (1 nm) for the indicated period. C/EBPβ expression levels were quantitated by RT-PCR. *P < 0.05 and **P < 0.001 and **b** MCF-7 cells were transferred to E_2_-free medium for 3 days, then MCF-7 and MCF-7:5C cells were treated with E_2_ for the indicated periods. C/EBPβ expression levels were quantitated by RT-PCR. *P < 0.05 and **P < 0.001 compared with control. **c** Knockdown of C/EBPβ by siRNA. MCF-7:5C cells were transfected with different C/EBPβ siRNAs for 72 h, and cells transfected with pooled or single scrambled siRNAs were used as controls. Cell lysates were harvested for immunoblot assay. **d**−**f**, **i**, **j** MCF-7:5C cells were transfected with C/EBPβ siRNA #8 for 72 h, and cells transfected with single scrambled siRNA were used as control. Next, transfected cells were treated with vehicle control or E_2_ (1 nm) for 72 h. NF-κB (**d**), TNFα (**e**), LTB (**f**), IL-6R (**i**), and IL-6 (**j**) expression levels were quantitated by RT-PCR. *P < 0.05 and **P < 0.001 compared with scrambled siRNA vehicle control. **g** MCF-7:5C cells were treated with vehicle control (0.1% DMSO) or rapamycin (20 nm) for 48 h. Phosphorylated p70S6 was measured using western blotting. **h** MCF-7:5C cells were treated with E_2_ (1 nm), rapamycin (20 nm), or a combination of them for 72 h. TNFα expression levels were quantitated by RT-PCR. **P < 0.001 compared with control. **k** MCF-7:5C cells were treated with E_2_ (1 nm), JSH-23 (20 µm), or a combination of them for 72 h. C/EBPβ expression levels were quantitated by RT-PCR. *P < 0.05 and **P < 0.001 compared with control

Cross-talk between NF-κB and C/EBPβ affects each other’s function^27^. We found that E_2_-deprived MCF-7:5C and MCF-7:2A had higher basal levels of C/EBPβ mRNA expression than did wild-type MCF-7 cells (Supplementary Fig. S2A). Treatment with the endoplasmic reticulum stress inducer tunicamycin (Tu) further increased expression of C/EBPβ in MCF-7:5C cells (Supplementary Fig. S2B). E_2_ started to upregulate C/EBPβ expression after 1 h of treatment (Fig. 3a) and continually elevated the expression of C/EBPβ in MCF-7:5C cells over levels in wild-type MCF-7 cells (Fig. 3b). This elevated C/EBPβ expression, induced by E_2_, was abolished by 4-hydroxytamoxifen (4-OHT), suggesting an ERα-dependent mechanism (Supplementary Fig. S2C). Treatment with estrogen dendrimer conjugate, a synthetic macromolecule that activates membrane-associated ERα^28^, could not activate C/EBPβ in MCF-7:5C cells (Supplementary Fig. S2D), confirming that elevation of C/EBPβ expression was mainly mediated by the nuclear ERα. We further used a specific siRNA to effectively knock down C/EBPβ protein expression in MCF-7:5C cells (Fig. 3c). This increased NF-κB mRNA expression, which was further elevated after E_2_-based treatment (Fig. 3d). In line with this result, expression of NF-κB-dependent gene TNFα was upregulated after knockdown of C/EBPβ (Fig. 3e). E_2_ was synergistic with C/EBPβ siRNA in increasing TNFα and LTB expression (Fig. 3e, f). Reduction of C/EBPβ expression by siRNA also elevated the expression of the apoptotic markers Bim and HMOX1 in MCF-7:5C cells, which increased more when C/EBPβ siRNA was combined with E_2_ (Supplementary Fig. S2E and S2F). Since the mTOR signal pathway has been observed to regulate the function of C/EBPβ in response to inflammation^29^, the mTOR inhibitor, rapamycin was used to effectively block phosphorylation of the downstream signal p70S6 (Fig. 3g). Similar to C/EBPβ siRNA, blocking mTOR signal was synergistic with E_2_ to increase the expression of NF-κB-dependent genes TNFα and LTB (Fig. 3h and Supplementary Fig. S2G). Additionally, C/EBPβ is well known to be a transcription factor that regulates IL-6 expression^30^. Of note, IL-6/IL-6R expression increased after knockdown of C/EBPβ and further with E_2_ treatment (Fig. 3i, j), indicating a suppressive effect on the IL-6/IL-6R pathway by C/EBPβ. Contrary to the repressive effects of C/EBPβ on NF-κB, inhibition of nuclear translocation of NF-κB reduced the basal expression of C/EBPβ and completely blocked the induction of C/EBPβ by E_2_ (Fig. 3k). This suggested a complex interaction existing between C/EBPβ and NF-κB in the settings of stress.

### Late activated NF-κB by E_2_ induces TNFα expression and causes apoptosis

**Fig. 4:**
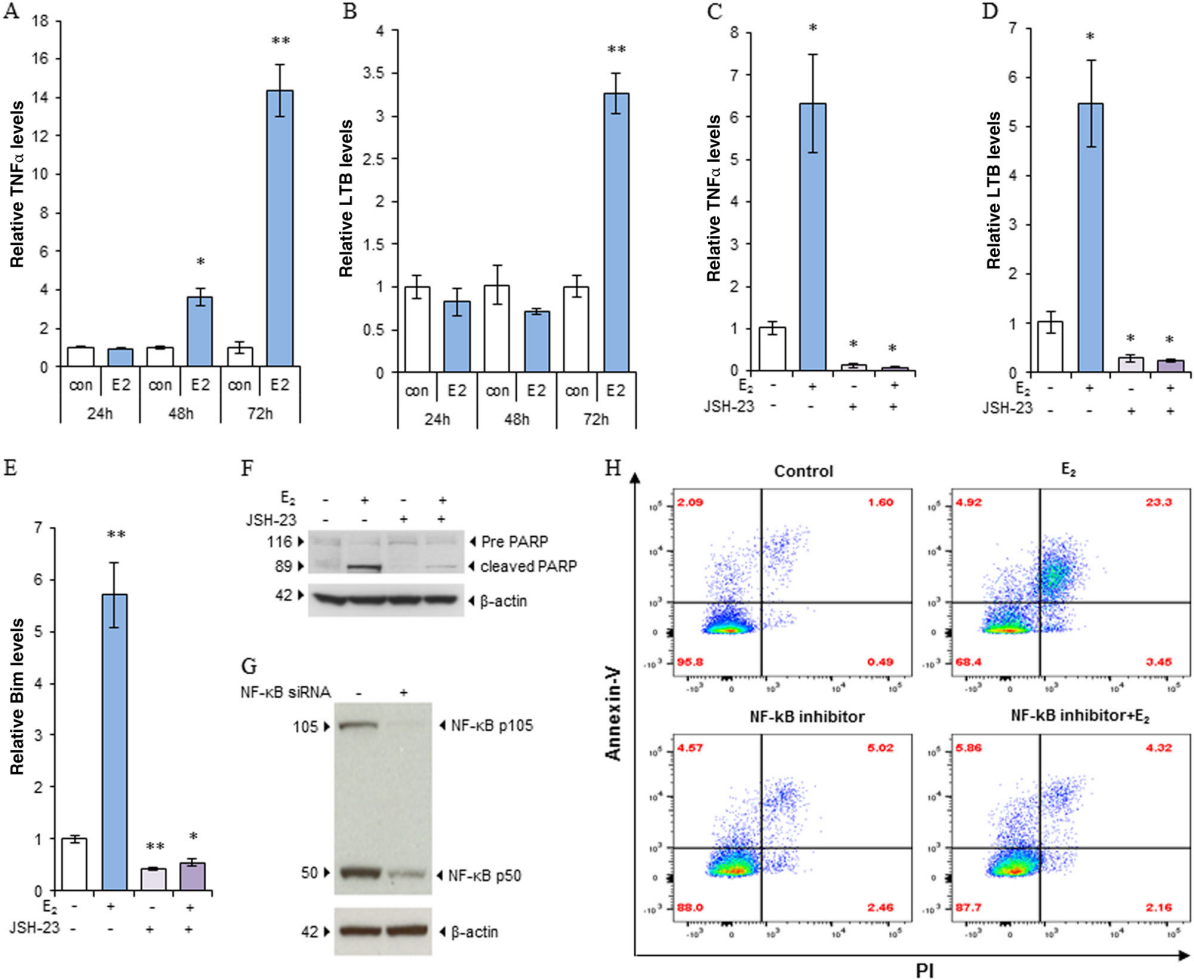
Late activated NF-κB is involved in the induction of TNFα expression and apoptosis by E_2_. **a**,** b**, MCF-7:5C cells were treated with vehicle control (0.1% EtOH) or E_2_ (1 nm) for the indicated periods. TNFα (**a**) and LTB (**b**) expression levels were quantitated by RT-PCR. *P < 0.05 and **P < 0.001 compared with control. **c**−**e** MCF-7:5C cells were treated with E_2_ (1 nm), JSH-23 (20 µm), or a combination of them for 72 h. TNFα (**c**), LTB (d), and Bim (e) expression levels were quantitated by RT-PCR. *P < 0.05 and **P < 0.001 compared with control. **f** MCF-7:5C cells were treated with E_2_ (1 nm), JSH-23 (20 µm), or a combination of them for 72 h. Cell lysates were harvested for western blotting using an anti-PARP antibody. **g** MCF-7:5C cells were transfected with a specific NF-κB siRNA for 72 h, using scrambled siRNA as control. Cell lysates were harvested for western blotting. **h** MCF-7:5C cells were treated with E_2_ (1 nm), JSH-23 (20 µm), or a combination of them for 72 h. Cells were harvested for Annexin V binding assay

The TNF family members are inflammatory factors induced by E_2_ to trigger apoptosis in two E_2_-deprived breast cancer cell lines^11, 18^. Our results demonstrated that treatment with E_2_ did not induce TNFα or LTB expression in MCF-7:5C cells within 24 h. However, E_2_ treatment began to increase the expression of TNFα and LTB after 48 h, peaking at 72 h (Fig. 4a, b). Other members of the TNF family, such as LTA, had similar dynamic changes in expression (Supplementary Fig. S3A). Treatment with the NF-κB inhibitor JSH-23 markedly reduced the basal expression of TNFα and LTB and effectively prevented E_2_ from inducing expression of TNFα (Fig. 4c), LTB (Fig. 4d), and LTA (Supplementary Fig. S3B). Similarly, E_2_ started to induce TNFα after 6 days of treatment and reached a peak after 12 days of exposure to E_2_ in another E_2_-deprived breast cancer cells, MCF-7:2A. JSH-23 mildly increased TNFα expression levels after 12 days administration, but it significantly blocked TNFα induction by E_2_ after combination treatment (Supplementary Fig. S3C). Additionally, inhibition of NF-κB activity reduced expression of the apoptotic marker Bim and completely blocked induction of Bim by E_2_ (Fig. 4e). Further biological experiments demonstrated that treatment with the NF-κB inhibitor effectively reduced cleavage of PARP activated by E_2_ (Fig. 4f). To confirm the roles of NF-κB in the induction of TNF family members, NF-κB was effectively knocked down by a specific siRNA (Fig. 4g). Consistently, depletion of NF-κB decreased the expression of TNFα and LTB and attenuated that of TNFα and LTB induced by E_2_ in MCF-7:5C cells (Supplementary Fig. S3D and S3E). More importantly, blockade of nuclear translocation of NF-κB by JSH-23 completely inhibited E_2_-induced apoptosis (Fig. 4h and Supplementary Fig. S3F). These results suggested that NF-κB participates in E_2_-induced apoptosis via mediation of the TNF family member-associated inflammatory factors.

### NF-κB is lately activated by PERK after E_2_ treatment

**Fig. 5:**
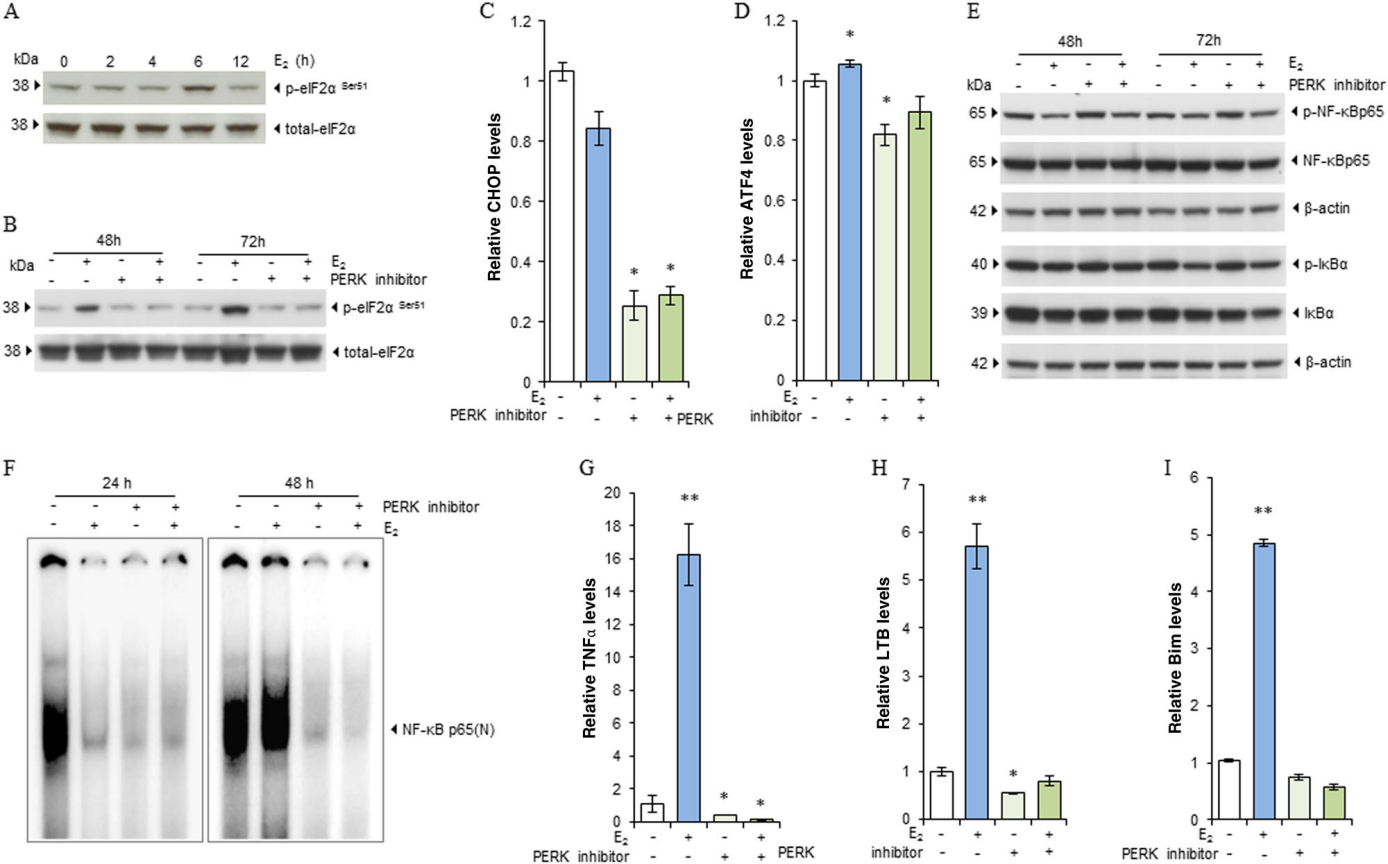
NF-κB is activated by PERK after E_2_ treatment. **a** MCF-7:5C cells were treated with vehicle control (0.1% EtOH) or E_2_ (1 nm) for the indicated periods. Phosphorylated and total eIF2α were measured using western blotting. **b** MCF-7:5C cells were treated with E_2_ (1 nm), PERK inhibitor (10 µm), or a combination of them for 48 and 72 h. Phosphorylated and total eIF2α were measured using western blotting. **c**,** d** MCF-7:5C cells were treated with E_2_ (1 nm), PERK inhibitor (10 µm), or a combination of them for 72 h. CHOP (**c**) and ATF4 (**d**) expression levels were quantitated by RT-PCR. *P < 0.05 compared with control. **e** Expression of phosphorylated NF-κB, total NF-κB, phosphorylated IκBα, and total IκBα were examined by western blotting. Protein cell lysates were the same as in **b**. **f** The PERK inhibitor blocked NF-κB DNA-binding. MCF-7:5C cells were treated with E_2_ (1 nm), PERK inhibitor (10 µm), or a combination of them for 24 and 48 h. Nuclear extracts were isolated for EMSA. **g**−**i** MCF-7:5C cells were treated with E_2_ (1 nm), PERK inhibitor (10 µm), or a combination of them for 72 h. TNFα (**g**), LTB (**h**), and Bim (**i**) mRNA expression levels were quantitated by RT-PCR. *P < 0.05 and **P < 0.001 compared with control

PERK activation is a key event in E_2_-induced apoptosis^11^. A question was raised whether PERK kinase could activate NF-κB after E_2_ treatment. E_2_ transiently activated the PERK downstream signal eIF2α in MCF-7:5C cells after 6 h of treatment (Fig. 5a) and continuously phosphorylated eIF2α after 24 h^11^. The phosphorylation of eIF2α could be blocked by a PERK inhibitor (Fig. 5b). Consistent with these findings, the downstream PERK genes, i.e. C/EBP-homologous protein (CHOP) and ATF4 expression were effectively blocked by the PERK inhibitor (Fig. 5c, d). Then, we examined the effects of the PERK inhibitor on the classic canonical pathways. Our results demonstrated that treatment with E_2_ weakly reduced the phosphorylation of NF-κB and IκBα, while inhibition of PERK kinase did not change the phosphorylation of NF-κB and IκBα (Fig. 5e). As for the total expression of the IκBα protein, E_2_ caused downregulation and could decrease IκBα amount further when combined with the PERK inhibitor (Fig. 5e). This result suggested that neither E_2_ nor PERK modulates the function of NF-κB through the canonical pathway. It is well known that NF-κB is a DNA-binding protein that transcriptionally regulates its target genes^31^. In the present study, MCF-7:5C cells had constitutively activated NF-κB exhibiting high basal levels of DNA binding. E_2_ significantly suppressed NF-κB DNA binding within 24 h, but this binding capacity returned after 48 h of exposure to E_2_ (Fig. 5f). Unexpectedly, the PERK inhibitor completely blocked the basal constitutive activation of NF-κB in MCF-7:5C cells and further activation by E_2_ (Fig. 5f). This inhibitor also effectively abolished induction of the NF-κB-dependent genes TNFα, LTB, Bim, and CXCR4 by E_2_ (Fig. 5g, i, and Supplementary Fig. S4A) and partially attenuated E_2_-induced IL-6 mRNA expression in MCF-7:5C cells (Supplementary Fig. S4B). Interestingly, inhibition of PERK kinase downregulated NF-κB mRNA expression (Supplementary Fig. S4C). In contrast, further depletion of PERK by an siRNA (Supplementary Fig. S4D) did not alter NF-κB mRNA expression (Supplementary Fig. S4E), indicating a transcriptional regulation of NF-κB via PERK kinase. Together, these findings suggested that PERK specifically modifies the nuclear function of NF-κB by increasing its DNA-binding capacity.

### PERK transcriptionally modulates the interaction between NF-κB and STAT3

**Fig. 6:**
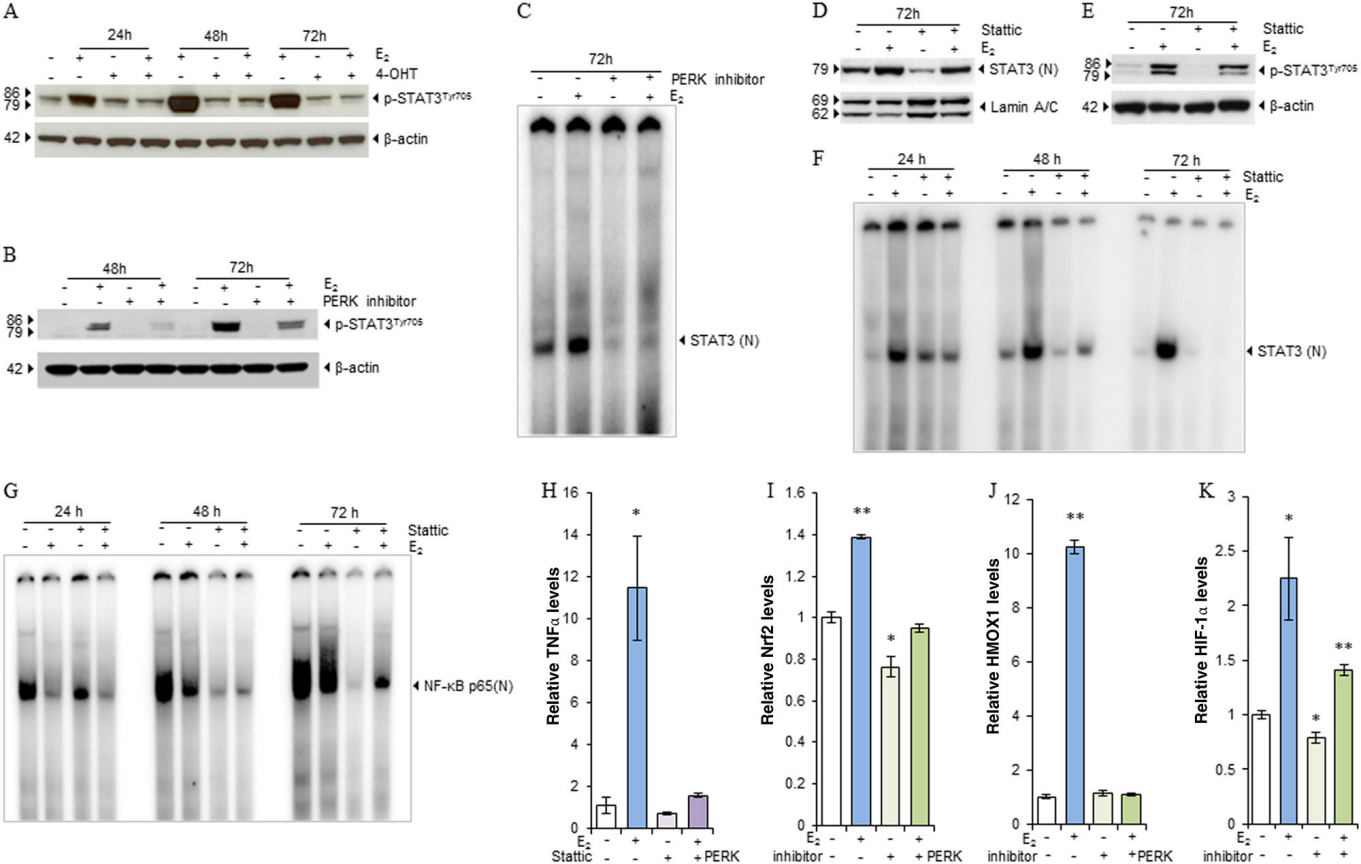
PERK modulates activation of NF-κB via STAT3. **a** MCF-7:5C cells were treated with E_2_ (1 nm), 4-OHT (1 µm), or a combination of them for 24, 48, 72 h. Phosphorylated STAT3 was measured using western blotting. **b** MCF-7:5C cells were treated with E_2_ (1 nm), PERK inhibitor (10 µm), or a combination of them for 48 and 72 h. Phosphorylated STAT3 was measured using western blotting. **c** The PERK inhibitor blocked STAT3 DNA-binding. MCF-7:5C cells were treated with E_2_ (1 nm), PERK inhibitor (10 µm), or a combination of them for 72 h. Nuclear extracts were isolated for EMSA. **d** MCF-7:5C cells were treated with E_2_ (1 nm), STAT3 inhibitor (5 µm), or a combination of them for 72 h. Nuclear extracts were isolated. STAT3 was examined by western blotting. **e** MCF-7:5C cells were treated with E_2_ (1 nm), STAT3 inhibitor (5 µm), or a combination of them for 72 h. Cell lysates were harvested. Phosphorylation of STAT3 was examined by Western blotting. **f**,** g** MCF-7:5C cells were treated with E_2_ (1 nm), STAT3 inhibitor (5 µm), or a combination of them for 24, 48, and 72 h. STAT3 (**f**) and NF-κB (**g**) DNA-binding were measured by EMSA. **h** MCF-7:5C cells were treated with E_2_ (1 nm), STAT3 inhibitor (5 µm), or a combination of them for 72 h. TNFα mRNA expression levels were quantitated by RT-PCR. *P < 0.05 compared with control. **i**−**k** MCF-7:5C cells were treated with E_2_ (1 nm), PERK inhibitor (10 µm), or a combination of them for 72 h. Nrf2 (**i**), HMOX1 (**j**), and HIF-1α (**k**) mRNA expression levels were quantitated by RT-PCR. *P < 0.05 and **P < 0.001 compared with control

**Fig. 7:**
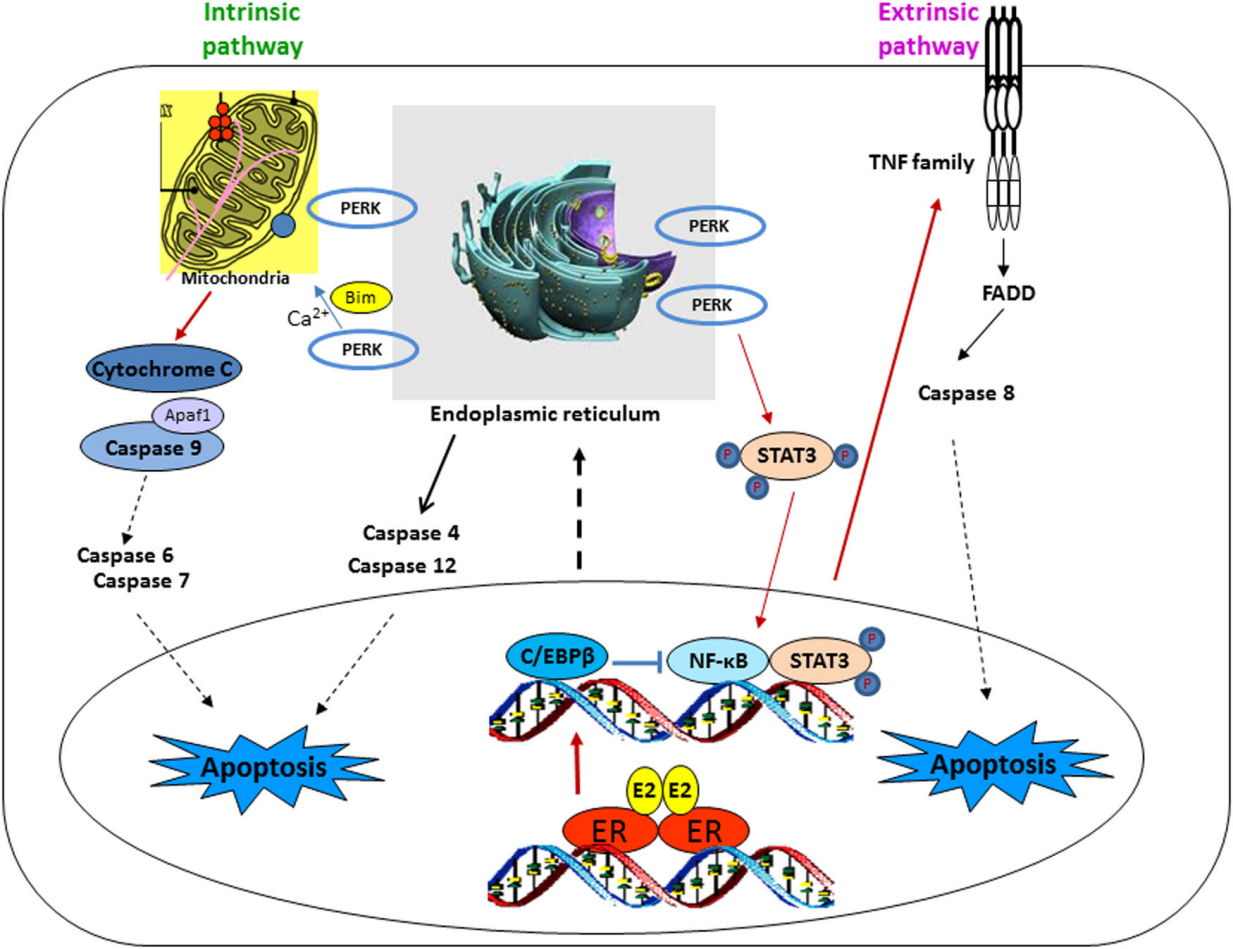
PERK is a key driver to activate STAT3/NF-κB/TNFα axis in E_2_-deprived breast cancer cells. Nuclear E_2_/ER preferentially activates C/EBPβ which can suppress NF-κB DNA binding. E_2_ also activates PERK in response to the accumulation of unfolded proteins in the endoplasmic reticulum. This stress kinase activates transcription factor STAT3 to increase NF-κB DNA binding which results in induction of TNF family members and associated apoptosis. Additionally, PERK regulates Nrf2 and serves as a contact site between endoplasmic reticulum and mitochondria or interacts with mitochondria via Bim/Ca^2+^ to activate oxidative stress-related apoptosis

Based on the results in Fig. 5 that PERK kinase mainly increased NF-κB DNA-binding (Fig. 5e, f), it indicates some other molecules are involved to mediate the responses between the endoplasmic reticulum and the nucleus. NF-κB and STAT3 are two closely related transcription factors in the regulation of inflammatory pathway^32^. To determine how PERK kinase modulates the nuclear function of NF-κB, we found that STAT3 was activated by E_2_ after 24 h of treatment, which was completely blocked by 4-OHT (Fig. 6a). Further inhibition of PERK kinase effectively blocked the phosphorylation of STAT3 (Fig. 6b) and STAT3 DNA-binding activity induced by E_2_ (Fig. 6c), demonstrating that STAT3 is a downstream signal of PERK. The STAT3 inhibitor, Stattic^33^, effectively blocked nuclear translocation of STAT3 (Fig. 6d), but almost did not affect the phosphorylation of STAT3 in MCF-7:5C cells (Fig. 6e). Notably, STAT3 DNA binding was increased by E_2_ in a time-dependent manner, and Stattic was far more effective to block STAT3 DNA binding than prevent nuclear translocation (Fig. 6e, f). Furthermore, the STAT3 inhibitor effectively blocked the DNA-binding activity of NF-κB (Fig. 6g) and the induction of NF-κB-dependent genes TNFα and LTB (Fig. 6h and Supplementary Fig. S5A and S5B). This finding suggested that STAT3 DNA binding is essential to affect the nuclear activation of NF-κB. In addition to regulating the function of STAT3 and NF-κB by PERK kinase, Nrf2 is a critical transcription factor that maintains redox homeostasis^34^. Treatment with E_2_ elevated Nrf2 expression in MCF-7:5C cells, whereas that with the PERK inhibitor reduced basal Nrf2 expression and effectively blocked the upregulation of Nrf2 expression by E_2_ (Fig. 6i). Consequently, inhibition of PERK kinase completely prevented expression of the oxidative stress indicator HMOX1 (Fig. 6j). A similar regulatory pattern was observed in stress-responsive transcription factor HIF-1α, which was upregulated by treatment with E_2_, but reduced by that with the PERK inhibitor. This inhibitor also blocked induction of HIF-1α expression by E_2_ (Fig. 6k). Since STAT3 acts as a downstream signal of PERK, inhibition of STAT3 also effectively blocked upregulation of Nrf2 and HMOX1 (Supplementary Fig. S5C and S5D), but not HIF-1α by E_2_ (Supplementary Fig. S5E). Intriguingly, the PERK inhibitor reduced expression of ERα-targeted genes pS2, c-Myc, and FOXO3 in MCF-7:5C cells (Supplementary Fig. S5F−S5H), despite the fact that E_2_ activates PERK via ERα^11^. Therefore, in addition to attenuating protein translation, PERK acts as a central regulator that modulates the function of multiple nuclear transcription factors in response to stress (Fig. 7).

## Discussion

Estrogen-induced apoptosis has clinical relevance for the treatment of aromatase inhibitor-resistant breast cancer. And it is a mechanistic interpretation for the decrease in breast cancer incidence and mortality of the E_2_ alone trial of the Women’s Health Initiative^6–8^. Endoplasmic reticulum stress precedes E_2_-induced apoptosis^12, 11^, which involves the activation of the three stress sensors with different functions in the modulation of cellular homeostasis^11–13^. Furthermore, PERK has been found as a pivotal stress sensor to regulate E_2_-induced apoptosis in E_2_-deprived breast cancer cells^11^. However, the mechanism whereby PERK interacts with transcription factors and integrally modulates E_2_-induced apoptosis is obscure. We have now deciphered the mechanism precisely. E_2_ suppresses the activation of NF-κB by preferentially upregulating C/EBPβ expression whereas PERK acts as a major driver to transcriptionally activate NF-κB/TNFα axis-linked apoptotic pathways in a delayed manner via STAT3.

A novel finding is how E_2_ modulates activation of NF-κB by alternate mechanisms to determine the fate of E_2_-deprived breast cancer cells. NF-κB is an inducible transcription factor that mediates numerous biological functions to regulate proliferation or apoptosis depending on the cellular context and inflammatory microenvironment^35–37^. Constitutive activation of NF-κB is one of the stress responses required for adaptation to long-term E_2_ deprivation, which initially acts as a growth signal in E_2_-deprived breast cancer cells as in other antiestrogen-resistant breast cancer cell lines^3, 38^. However, E_2_ exposure completely alters the biological functions of NF-κB in E_2_-deprived breast cancer cells. Nuclear ERα, but not membrane-associated ERα, is known to be activated by E_2_ to induce apoptosis^11^, which preferentially elevates expression of the lipid metabolism-associated transcription factor C/EBPβ^39^ to suppress the activation of NF-κB. This repressive potential of E_2_ is enough to compete with a strong inducer, TNFα, to prevent NF-κB activation. In line with our findings, Hayakawa et al.^27^ demonstrated a suppressive interaction between C/EBPβ and NF-κB in mesangial cells under endoplasmic reticulum stress. C/EBPβ and NF-κB belong to distinct families of transcription factors that have functional and physical associations between their DNA-binding domains or through protein−protein interactions^40, 41^. Although mechanisms whereby C/EBPβ suppresses NF-κB are unclear, it is very likely that predominant activation of C/EBPβ in settings of endoplasmic reticulum stress precedes NF-κB to occupy DNA-binding regions or form protein complexes with the majority of NF-κB, subsequently preventing NF-κB DNA binding. Furthermore, C/EBPβ is an important adipogenic transcription factor with a function that is closely associated with the mTOR transduction pathway that develops in response to stresses^29, 42^. The phospholipid-associated pathways PI3K/Akt/mTOR are activated early by E_2_ in MCF-7:5C cells^13^, whereas these signals are degraded by IRE1 and ATF-6-mediated ERAD after 48 h of E_2_ action^13^. Therefore, attenuation of the mTOR signal by UPR might dissociate the inhibitory interactions between C/EBPβ and NF-κB, which results in the activation of NF-κB at a later time.

The mechanism by which PERK kinase modulates NF-κB DNA binding provides an in-depth understanding of E_2_-induced apoptosis in E_2_-deprived breast cancer cells. A central biological function of PERK is to reduce the protein burden in the endoplasmic reticulum by activating eIF2α kinase^11, 16^. Deng et al.^25^ reported that eIF2α activates NF-κB by decreasing levels of the NF-κB inhibitor, IκBα. By contrast, our results demonstrate that inhibition of eIF2α activity by a PERK inhibitor decreases IκBα expression which further blocks NF-κB activity. These data suggest that eIF2α phosphorylation is not required to activate NF-κB by PERK. Consistent with our results, Cullinan et al.^34^ found that PERK regulates nuclear translocation of the redox homeostasis modulator Nrf2 without requirement of eIF2α phosphorylation. Our evidence also indicates that PERK does not regulate the canonical IκBα signal pathway to affect NF-κB DNA binding. Of note, STAT3 is identified as a target transcription factor that is regulated by PERK to activate NF-κB DNA binding and NF-κB-dependent genes. Furthermore, our finding demonstrates that STAT3 DNA binding, rather than phosphorylation can significantly affect NF-κB DNA binding, indicating an essential interaction between STAT3 and NF-κB in DNA. There are many signals that might be involved in the modulation of STAT3 by PERK. For instance, PERK activates cytokine IL-6 which is a strong inducer for STAT3^43^. Additionally, PERK-dependent Janus kinase 1 (JAK1) and interaction with tyrosine kinase c-Src may also regulate the function of STAT3^11, 24, 44^. It is worthy to mention here that both STAT3 and NF-κB are important mediators for PERK to regulate Nrf2, which is a substrate of PERK^34, 45^, thereby modulating oxidative stress in the mitochondria. Recently, a novel function of PERK was described^46, 47^ as a structural tether to increase the proximity of contact sites between the endoplasmic reticulum and mitochondria, which may facilitate oxidative stress. Nevertheless, how PERK actually modulates these stress-responsive transcription factors is various, which depends on the extent of PERK activation and different cell context^48^. All of these findings support the conclusion that PERK plays a central role to convey both adaptive and apoptotic signals from the endoplasmic reticulum to the nucleus^49^.

Our results also demonstrate that NF-κB widely interacts with multiple transcription factors, such as ERα, C/EBPβ, and STAT3 to modulate stress responses, inflammatory responses, and apoptosis. Although the NF-κB/TNFα axis plays an important role in the E_2_-induced apoptosis in MCF-7:5C cells, the function of many other TNF family members are needed to further investigate. Even MCF-7:5C and MCF-7:2A cells both are derived from the same parental MCF-7 cells. MCF-7:5C cells have higher basal levels of TNFα, LTA, and LTB than those in MCF-7:2A cells; while MCF-7:2A cells have been found to express higher levels of TNFRSF18, TNFRSF19, and TNFRSF8 than those in MCF-7:5C cells^18^. How NF-κB modulates these TNF family members in MCF-7:2A cells remains unclear. It is worthy to make a note that our results obtain from limited E_2_-deprived breast cancer cell lines. Currently, all published E_2_-induced apoptosis in vitro are observed in MCF-7-derived E_2_-deprived breast cancer cell lines^4, 5^. ERα is the initial site for E_2_ to induce apoptosis^11^. There are four wild-type ERα-positive breast cancer cell lines (MCF-7, T47D, ZR-75-1, and BT-474) available for laboratory research^50^. Among them, MCF-7 and T47D are two representative ERα-positive breast cancer cell lines being widely used for research. They have distinct alterations in ERα expression after E_2_-deprivation^50^. ERα expression levels are increased in MCF-7-derived E_2_-deprived breast cancer cell lines^4, 5^, whereas ERα expression levels are decreased to undetectable in T47D-derived E_2_-deprived breast cancer cell line: T47D:C42^51^. Thus, T47D:C42 cells have no response to E_2_ after E_2_-deprivation^51^. In our group, two new E_2_-deprived breast cancer cell lines derived from ZR-75-1 and BT-474 are in developing which will expand cell lines for further investigation of E_2_-induced apoptosis in breast cancer.

Collectively, the PERK/STAT3/NF-κB/TNFα axis is central to ensure E_2_-induced apoptosis mediated via the endoplasmic reticulum. This in turn facilitates oxidative stress within mitochondria and activates inflammatory responses, leading to the secretion of numerous cytokines^11, 52^. All of these factors form an inflammatory microenvironment to integrally regulate the biological function of transcription factors and decide the fate of cells after E_2_ exposure^11, 12^. The key role of PERK in the modulation of apoptosis suggests that accumulation of unfolded proteins is an initial burden generated by E_2_ in the endoplasmic reticulum. Consequently, determining how E_2_ produces unfolded proteins to trigger endoplasmic reticulum stress-associated apoptosis is essential to find the mechanisms underlying E_2_-induced apoptosis. Our recent observations demonstrate that E_2_ preferentially and consistently activates some transcription factors with short half-lives, such as c-Fos in E_2_-deprived cells. These have high potential to cause an accumulation of aberrant unfolded proteins in the endoplasmic reticulum. A rigorous investigation of the apoptosis triggering mechanism in human cancer cell models provides valuable insight into a vulnerability of endocrine resistant cancer. Future application of this knowledge will aid development of treatments to increase breast cancer patient survival.

## Materials and methods

### Materials

Estrogen and rapamycin were purchased from Sigma-Aldrich. The NF-κB inhibitor, JSH-23 was purchased from CalBiochem. The PERK inhibitor, GSK797800 was obtained from Toronto Research Chemicals. The STAT3 inhibitor, Stattic was purchased from Tocris. For western blotting, antibodies against NF-κB, phosphorylated-NF-κB, phosphorylated-IκBα, total-IκBα, phosphorylated-eIF2α, total-eIF2α, PERK, and PARP were purchased from Cell Signaling Technology. NF-κB, C/EBPβ, PERK and scrambled siRNAs were obtained from GE Dharmacon.

### Cell culture conditions and cell line validation

MCF-7:WS8 cells were clonally selected from their parental counterpart MCF-7 (obtained from Dean Edwards, San Antonio, TX) for sensitivity to growth stimulation by E_2_, which were used in all experiments indicating MCF-7. MCF-7 cells were maintained in phenol red containing RPMI 1640 medium supplemented with 10% fetal bovine serum. MCF-7:5C and MCF-7:2A cells were cloned from E_2_-deprived MCF-7 cells and maintained in phenol red-free RPMI 1640 supplemented with 10% dextran-coated charcoal-stripped fetal bovine serum. Three cell lines were validated by a single nucleotide polymorphism (SNP) based assay at MD Anderson’s Characterized Cell Line Core (CCLC) on January 31, 2017. The SNP ID pattern of three cell lines is consistent with the report of the CCLC standard MCF-7 cells (Supplementary Table S1).

### Annexin V analysis of apoptosis

A FITC Annexin V Detection Kit I (BD Pharmingen) was used to quantify apoptosis of MCF-7:5C cells through flow cytometry according to the manufacturer’s instructions. In brief, MCF-7:5C cells were seeded in 10-cm dishes. The next day, the cells were treated with different compounds for different periods. Cells were suspended in 1× binding buffer and 1 × 10^5^ cells were stained simultaneously with FITC-labeled annexin V (FL1-H) and propidium iodide (FL2-H). The cells were analyzed using a FACSort flow cytometer (Becton Dickinson).

### Subcellular fractionation and immunoblotting

Cytosolic extract of MCF-7:5C cells was prepared using cell lysis buffer. Cytosolic cell lysates were spun at 12,000 rpm for 2 min, and supernatants were collected in separate eppendorf tubes. Nuclear extracts were prepared using a nuclear extraction. Nuclear cell lysates were spun at 14,000 rpm for 10 min and supernatants were collected and kept at −80 °C. Total proteins were extracted in cell lysis buffer (Cell Signaling Technology) supplemented with Protease Inhibitor Cocktail (Roche) and Phosphatase Inhibitor Cocktail Set I and Set II (CalBiochem). Immunoblotting was performed as described previously^11^.

### Electrophoretic mobility shift assays

To assess the nuclear activation of NF-kB and STAT3 in MCF-7:5C cells, EMSA of nuclear extracts from treated- and untreated-cells was performed as described previously^53^. In brief, MCF-7:5C cells (2 × 10^6^/mL) were treated with E_2_, a PERK inhibitor, or a STAT3 inhibitor for different periods. Nuclear extracts from the cells were incubated with a ^32^P end-labeled NF-κB oligonucleotide (15 μg of protein with 16 fmol of DNA) (5′-TTGTTACAAGGGACTTTCCGCTGGGGACTTTCCAGGGAGGCGTGG-3′, with NF-κB-binding sites) or two ^32^P-labeled high-affinity sis-inducible element (hSIE) STAT3 probes (5′-CTTCATTTCCCGTAAATCCCTAAAGCT-3′ and 5′-AGCTTTAGGGATTTACGGGAAATGA-3′)^54^ for 30 min at 37 °C. The resulting protein-DNA complex was separated from free oligonucleotides on 6.6% native polyacrylamide gels. The dried gels were visualized via exposure on X-ray films, and radioactive bands were analyzed.

### Quantitative real-time reverse transcription-PCR

Total RNA isolated from cells using an RNeasy Micro kit (Qiagen) was converted to first-strand cDNA using a high-capacity cDNA reverse transcription kit (Applied Biosystem). Quantitative real-time PCR assays were performed with SYBR Green PCR Master Mix (Applied Biosystems) and a QuantStudio 6 Flex real-time PCR System (Applied Biosystems). All primers were synthesized in Integrated DNA Technologies. All data were normalized by 36B4.

### Statistical analysis

All values are reported as means ± SEM. Statistical comparisons were carried out using a two-tailed Student’s *t*-test. Results were considered statistically significant if the *P*-value was less than 0.05.

## Supplementary information

Validation of three cell lines
The function of NF-κB in three breast cancer cell lines
Interaction between NF-κB and C/EBPβ in MCF-7:5C cells
Regulation of NF-κB-dependent genes by treatment with E2
Regulation of NF-κB-associated genes by PERK
Regulation of NF-κB-associated genes or other transcription factors by STAT3 and PERK
