# Supplementary figures and images for: Modulation of nuclear factor-kappa B activation by the endoplasmic reticulum stress sensor PERK to mediate estrogen-induced apoptosis in breast cancer cells
Source: Cell Death Discov. 2018 Feb 12;4:15. doi: 10.1038/s41420-017-0012-7 (PMC5841410; doi:10.1038/s41420-017-0012-7)

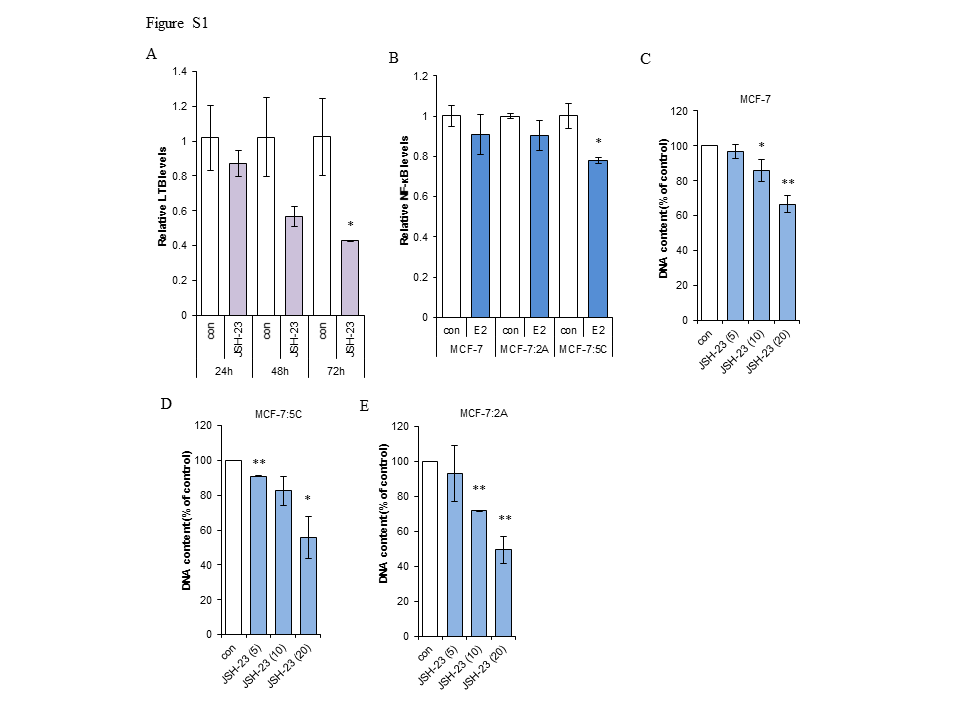

Supplement: Supplementary file 2 — The function of NF-κB in three breast cancer cell lines [file 41420_2017_12_MOESM2_ESM.tif]

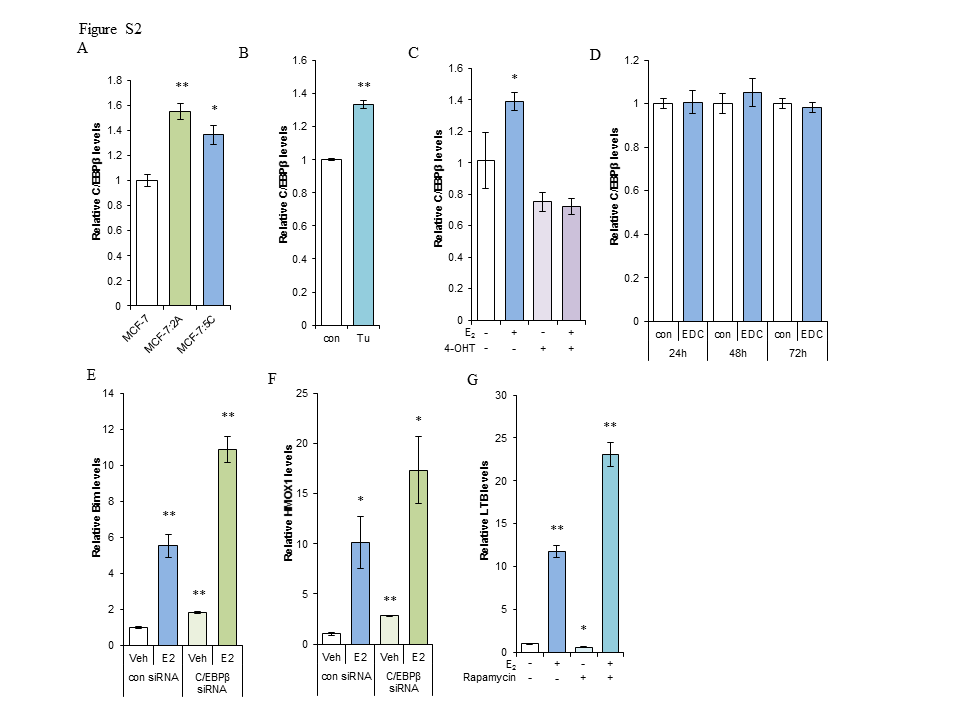

Supplement: Supplementary file 3 — Interaction between NF-κB and C/EBPβ in MCF-7:5C cells [file 41420_2017_12_MOESM3_ESM.tif]

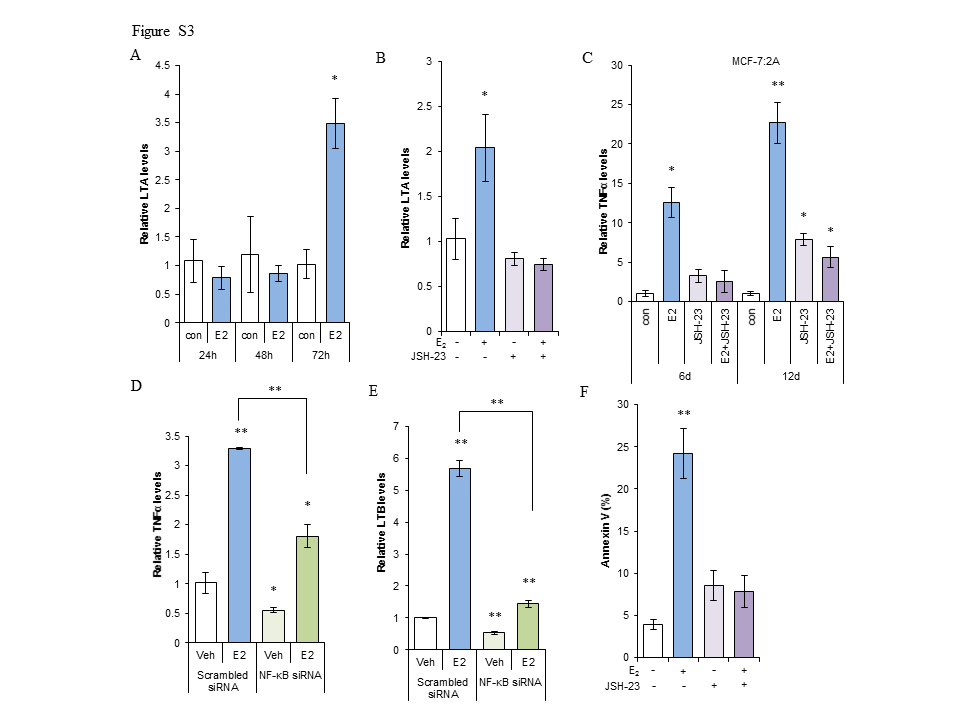

Supplement: Supplementary file 4 — Regulation of NF-κB-dependent genes by treatment with E2 [file 41420_2017_12_MOESM4_ESM.tif]

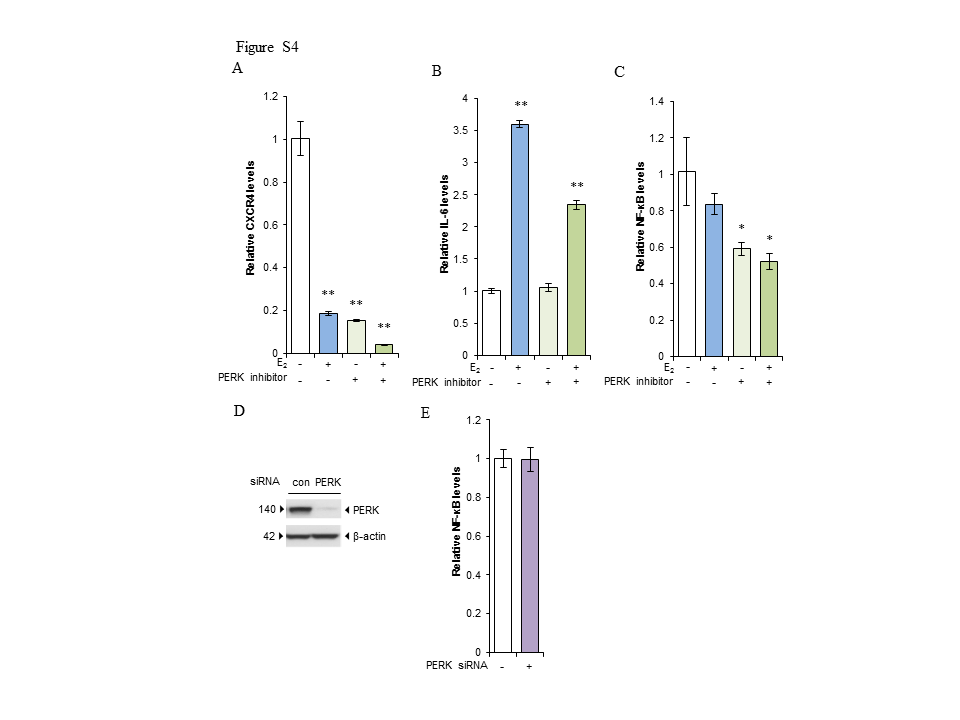

Supplement: Supplementary file 5 — Regulation of NF-κB-associated genes by PERK [file 41420_2017_12_MOESM5_ESM.tif]

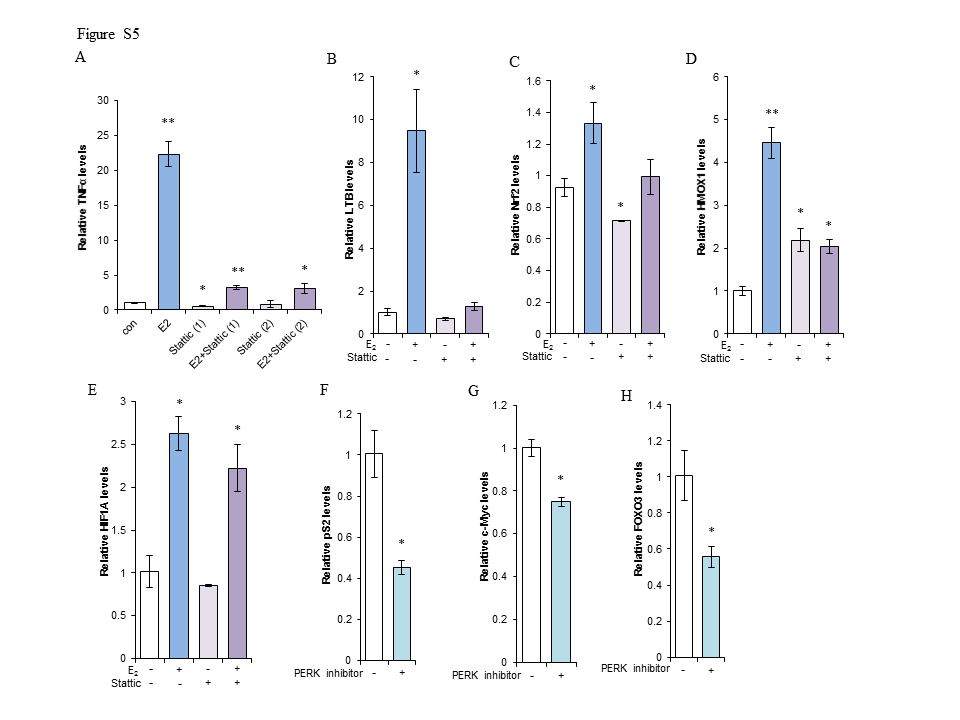

Supplement: Supplementary file 6 — Regulation of NF-κB-associated genes or other transcription factors by STAT3 and PERK [file 41420_2017_12_MOESM6_ESM.tif]
